# Supplementary material for: Baicalein Activates Parkin-Dependent Mitophagy through NDP52 and OPTN
Source: Cells. 2022 Mar 28;11(7):1132. doi: 10.3390/cells11071132 (PMC8997844; doi:10.3390/cells11071132)
Supplement: Supplementary file 1 [file cells-11-01132-s001.zip › cells-1608165-supplementary.pdf]

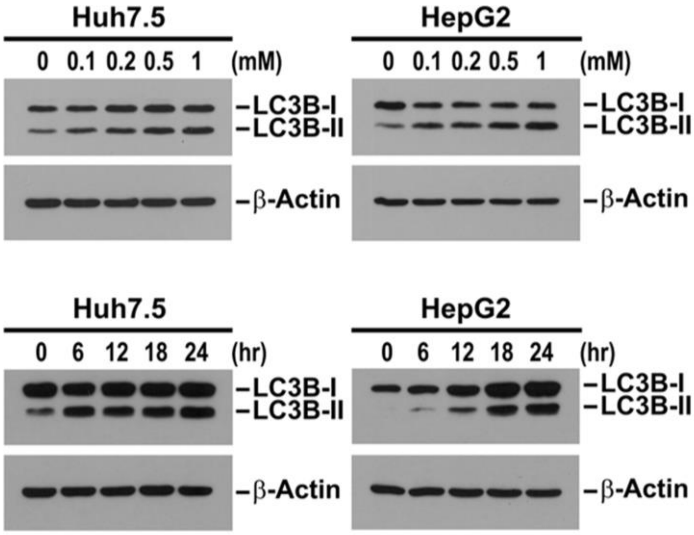

Supplemental Figure S1. Induction of LC3B-II formation by baicalein: Top row: Huh7.5 and HepG2 cells were treated with baicalein at the indicated concentrations. Twelve hours later, the cells were harvested and analyzed for protein expression by western blotting. Bottom row: Huh7.5 and HepG2 cells were treated with 0.5 mM baicalein and harvested at different times for use in the western blot analysis.

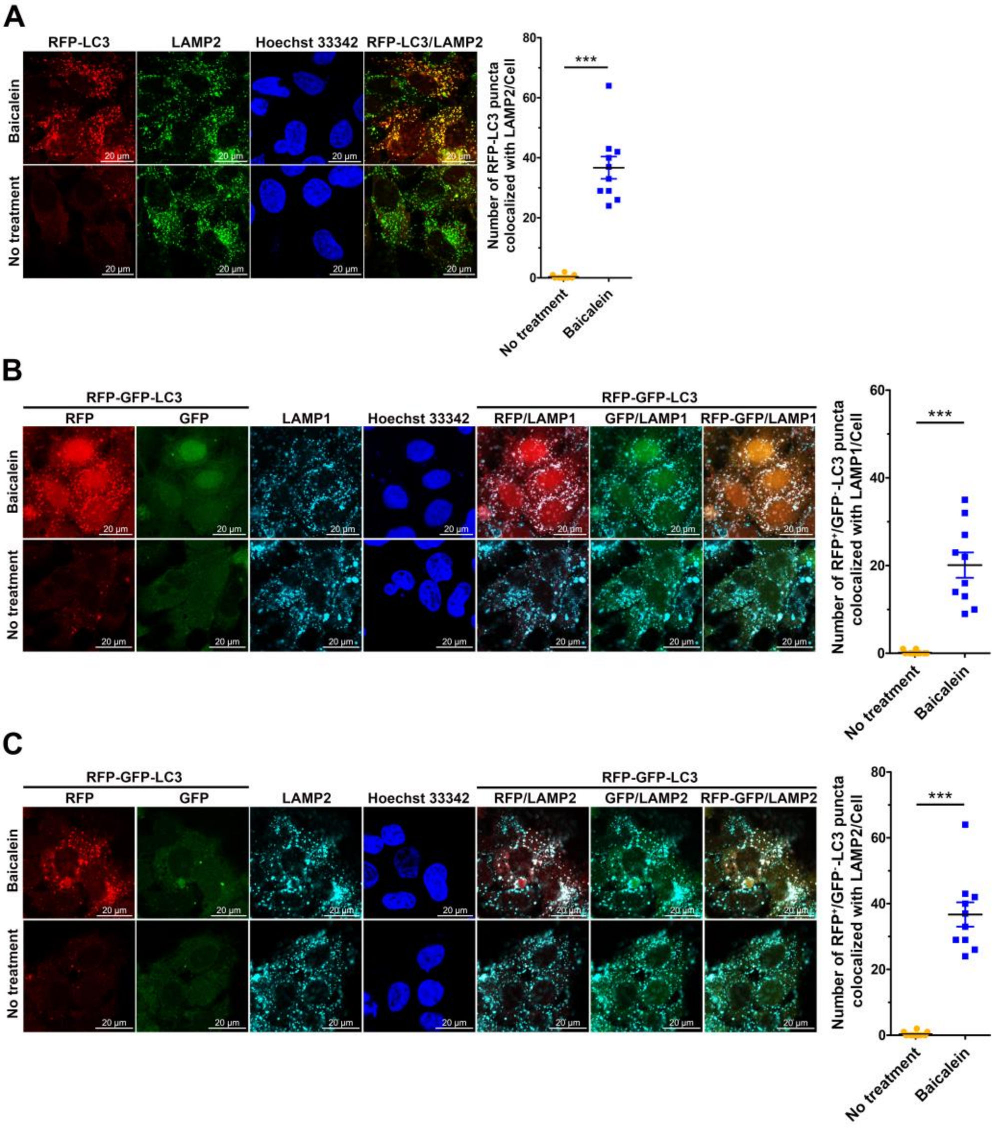

Supplemental Figure S2. Activation of autophagosome-lysosome fusion in baicalein-treated cells: **(A)** Huh7/RFP-LC3 cells were treated with or without 0.5 mM baicalein. Four hours later, the cells were fixed and immunostained with LAMP2 (green) and Hoechst 33342 (blue). The number of RFP-LC3 puncta colocalized with LAMP2 was quantified. The data represent the means ± SEM (n=10, ***P<0.001). **(B)** The baicalein-treated and untreated Huh7/RFP-GFP-LC3 cells shown in Figure 2B were fixed, immunostained for LAMP1 and nuclei (Hoechst 33342), and analyzed by confocal microscopy. The degree of colocalization between LAMP1 and RFP^+/GFP^- autolysosomes was quantified. The data represent the means ± SEM (n=10, ***P<0.001). **(C)** Huh7/RFP-GFP-LC3 cells were treated with baicalein as described in **(B)**, fixed, immunostained for LAMP2 and nuclei (Hoechst 33342), and assessed using confocal microscopy. The number of LAMP2 signals colocalized with RFP^+/GFP^- autolysosomes was quantified, and the data represent the means ± SEM (n=10, ***P<0.001).

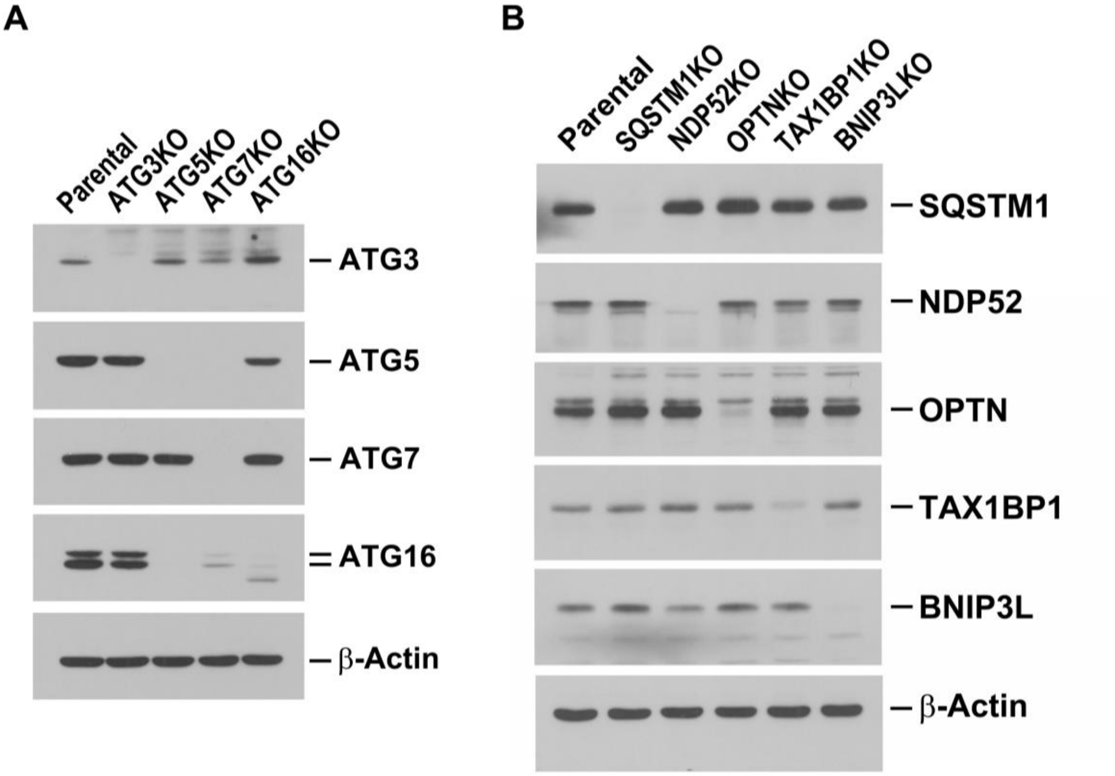

Supplemental Figure S3. The effect of knocking out ATG gene expression on autophagosome maturation and cargo receptors for selective autophagy: **(A)** ATG3KO, ATG5KO, ATG7KO, and ATG16 KO Huh7 cells were established by CRISPR/Cas9 gene editing as described in "Materials and Methods". **(B)** Individual Huh7 cell lines with SQSTM1, NDP52, OPTN, TAX1BP1, or BNIP3L knocked out were generated by CRISPR/Cas9 gene editing, as shown in the "Materials and Methods". Parental Huh7 cells were transduced with lentiviruses packaged with the Cas9/sgRNA transfer vector lacking a single guide RNA (sgRNA) insert. The parental cells and individual KO cell lines were harvested, and protein expression was determined by western blotting.

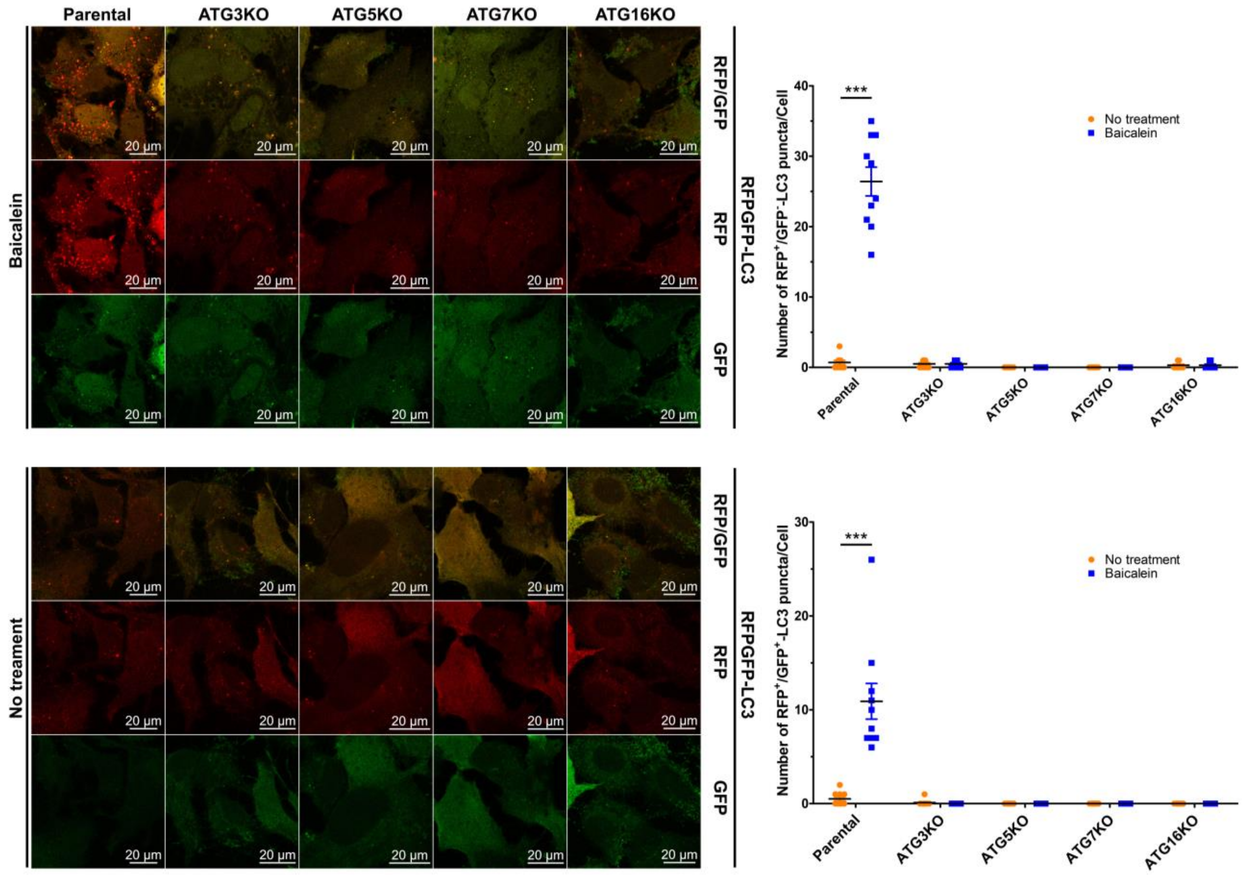

Supplemental Figure S4. Requirement of the UBL conjugation of ATGs for baicalein-activated autophagy: Lentiviruses harboring RFP-GFP-LC3 were transduced into parental and individual ATG-KO cell lines. Then, the cells were cultured in the presence or absence of 0.5 mM baicalein for four hours. The cells were fixed, stained with Hoechst 33342, and observed by confocal microscopy. The numbers of RFP^+/GFP^- puncta (autolysosomes) and RFP^+/GFP^+ puncta (autophagosomes) in parental cells and each ATG-KO cell line were quantified. The data represent the means ± SEM (n=10, ***P<0.001).

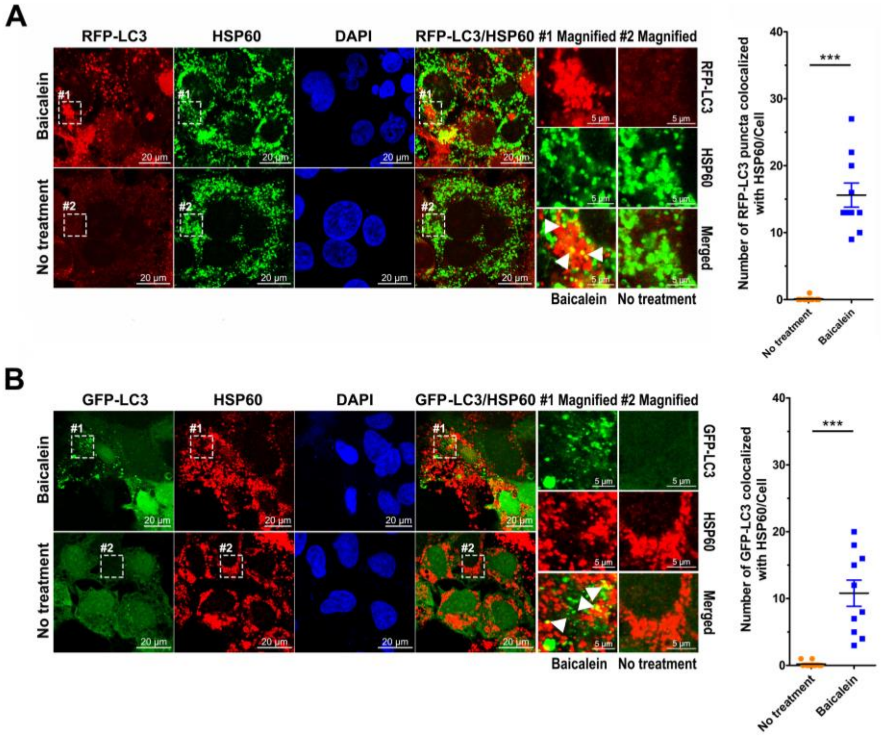

Supplemental Figure S5. Colocalization of mitochondria with baicalein-induced autophagic vacuoles: **(A)** Huh7/RFP-LC3 cells were treated with or without 0.5 mM baicalein for four hours. Then, the cells were fixed, immunostained for HSP60 (green) and nuclei by DAPI (blue), and analyzed by confocal microscopy. **(B)** Treatment with baicalein, immunostaining for HSP60 and nuclei, and confocal microscopy analysis of the Huh7/GFP-LC3 cells were performed as described in **(A)**. The number of RFP-LC3 puncta colocalized with HSP60 **(A)** and the number of GFP-LC3 colocalized with HSP60 **(B)** were quantified. The data represent the means ± SEM (n=10, ***P<0.001). In each panel, magnified field-1 and magnified field-2 show enlarged images of the areas in white dashed boxes 1 and 2 in the images of baicalein-treated and untreated cells, respectively. The white arrowheads in magnified field-1 indicate the colocalized signal.

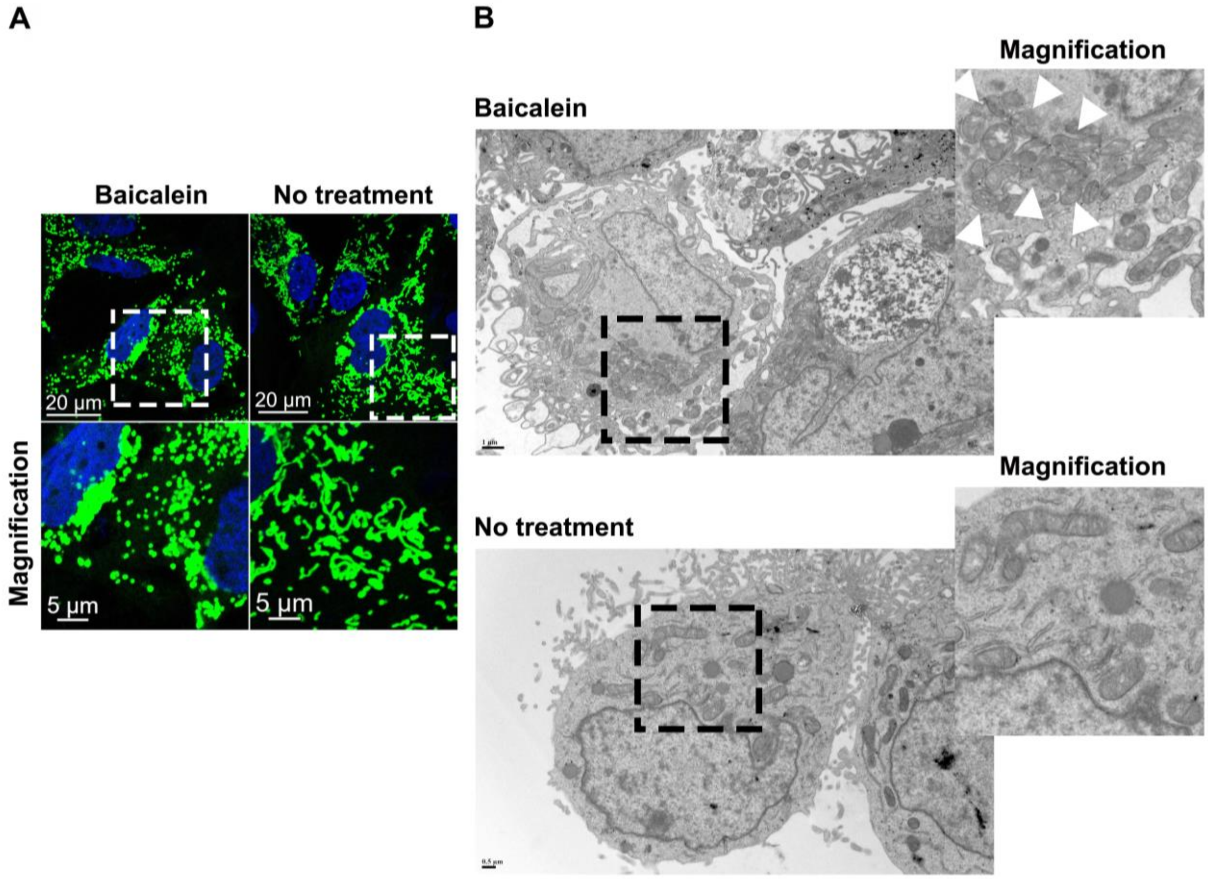

Supplemental Figure S6. Induced mitochondrial fragmentation and aggregation by baicalein: **(A)** Huh7/Mito-GFP cells were treated with or without 0.5 mM baicalein for four hours. Then, the cells were fixed and analyzed by confocal microscopy. The magnified images represent the enlargement of the white dashed boxes. **(B)** Huh7 cells treated with baicalein as described in **(A)** were processed for TEM analysis. The magnified images in the right-upper corner show the enlarged black dashed boxes. The white arrowheads indicate the clustering of fragmented mitochondria.

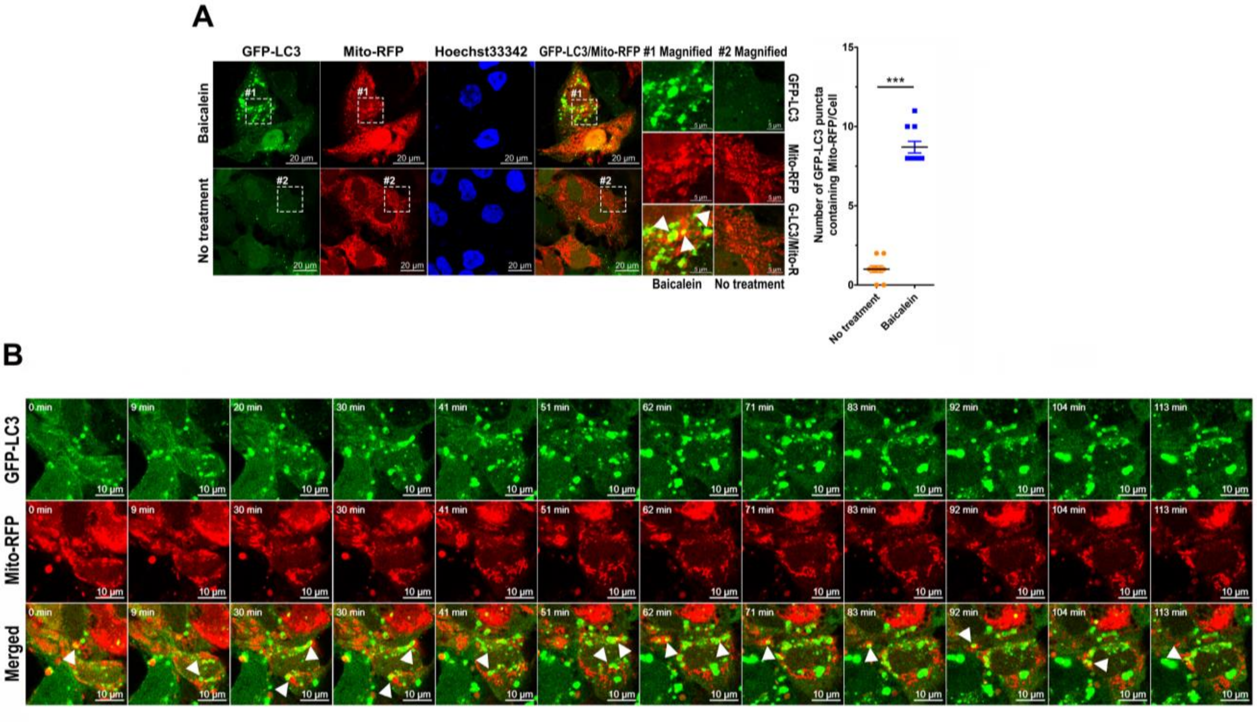

Supplemental Figure S7. Dynamics of sequestration of mitochondria in baicalein-activated autophagy: **(A)** Huh7/GFP-LC3/Mito-RFP cells established by lentivirus transduction as described in "Materials and Methods" were treated with baicalein and analyzed by confocal microscopy as described in Figure 5A The sequestration of Mito-RFP within GFP-LC3 puncta is indicated by white arrowheads. The degree of colocalization between Mito-RFP-labeled mitochondria and GFP-LC3 puncta was quantified. The data represent the means ± SEM (n=10, ***P<0.001). Magnified field-1 and magnified field-2 show the enlarged images of the areas in white dashed boxes 1 and 2 in the images of baicalein-treated and untreated cells, respectively. The white arrowheads in magnified field-1 indicate the colocalized signal. **(B)** Selected live imaging frames showing 0.5 mM baicalein-treated Huh7/GFP-LC3/Mito-RFP cells were obtained from Supplemental Video S5.

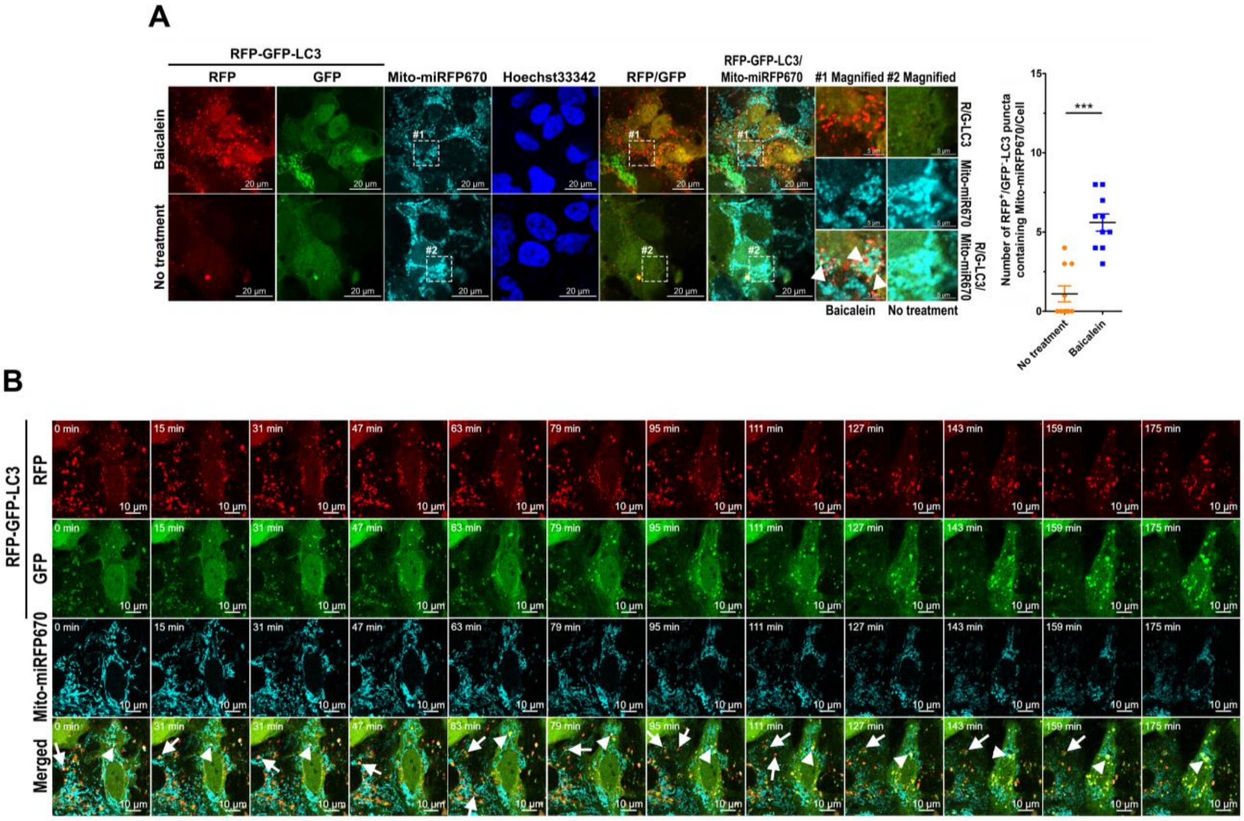

Supplemental Figure S8. Dynamics of mitochondrial engulfment by autophagy in baicalein-treated cells: **(A)** Lentiviruses harboring RFP-GFP-LC3 and Mito-miRFP670 were transduced into Huh7 cells to establish Huh7/RFP-GFP-LC3/Mito-miRFP670 cells. Then, the cells were cultured in the presence or absence of 0.5 mM baicalein, fixed and stained with Hoechst 33342, and analyzed by confocal microscopy. The degree of colocalization between Mito-miRFP670-labeled mitochondria and RFP^+/GFP^- LC3 puncta was quantified. The data represent the means ± SEM (n=10, ***P<0.001). Magnified field-1 and magnified field-2, respectively show the enlarged images of the areas in white dashed boxes 1 and 2 in the images of baicalein-treated and untreated cells. The white arrowheads in the magnified field-1 indicate the colocalized signal. **(B)** Selected live imaging frames showing 0.5 mM baicalein-treated Huh7/GFP-LC3/Mito-RFP cells. The sequestration of Mito-miRFP670 in RFP^+/GFP^+ and RFP^+/GFP^- LC3 puncta-labeled autophagosomes and autolysosomes is indicated by white arrowheads and white arrows. All frames in sequence are shown in Supplemental Video S6.

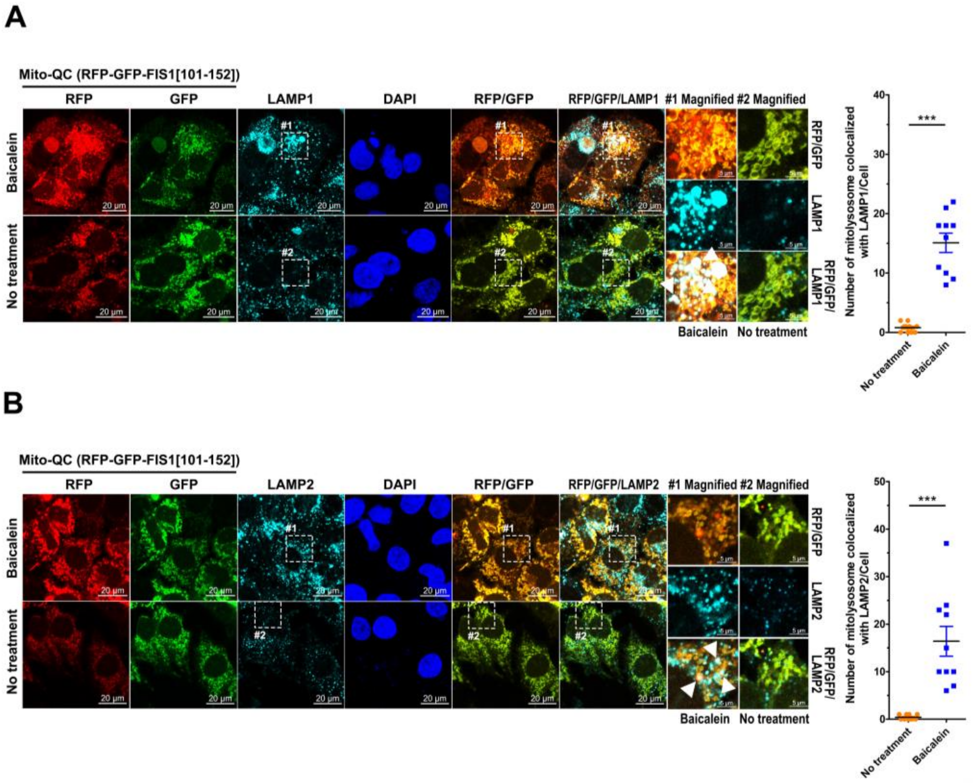

Supplemental Figure S9. Mitolysosome formation in baicalein-treated cells: **(A)** Huh7 cells stably expressing Mito-QC (Huh7/Mito-QC) were established by lentiviral gene delivery as described in "Materials and Methods". The cells were treated with or without 0.5 mM baicalein for four hours, fixed, immunostained with LAMP1 and DAPI (nuclei), and analyzed by confocal microscopy. **(B)** The baicalein-treated Huh7/Mito-QC cells described in **(A)** were fixed and stained for visualizing LAMP2 and nuclei (DAPI). The immunostained cells were analyzed by confocal microscopy. The number of colocalizations of LAMP1 with mitolysosomes in **(A)** and the number of colocalizations of LAMP2 with mitolysosomes in **(B)** were quantified. The data represent the mean ± SEM (n=10, ***P<0.001). Magnified field-1 and magnified field-2, respectively show the enlarged images of the areas in white dashed boxes 1 and 2 in the images of baicalein-treated and untreated cells in each panel. The white arrowheads in the magnified field-1 of **(A)** and **(B)** indicate the colocalized signal.

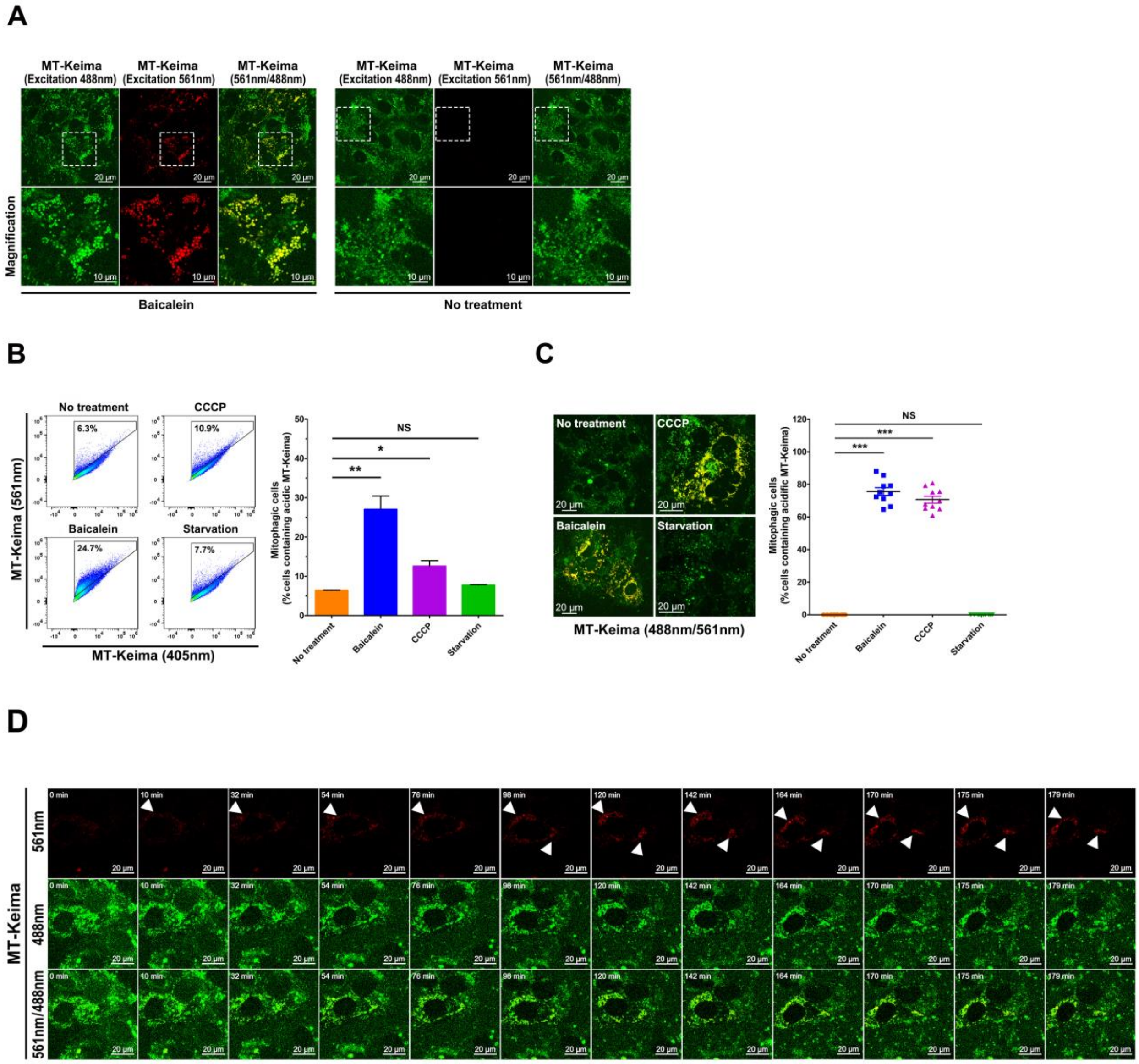

Supplemental Figure S10. Mitophagic degradation in baicalein-treated cells: **(A)** Huh7/MT-Keima cells established by lentivirus transduction as described in the "Materials and Methods" were treated with or without 0.5 mM baicalein for four hours and analyzed based on short (488 nm) and long (561 nm) excitation wavelengths by confocal microscopy. The images in the bottom row show the areas in the white dashed boxes at higher magnification. **(B)** Huh7/MT-Keima cells treated with 0.5 mM baicalein for four hours, treated with CCCP for two hours, and nutrient-starved for two hours were subjected to FACS analysis of MT-Keima expression with excitation wavelengths of 405 nm and 561 nm. Representative data from three independent experiments are shown in the left panel. The percentage of mitophagic cells in each sample was quantified by FlowJo software and is shown in the right panel. The data represent the means ± SEM (n=10, *P<0.05, **P<0.01, NS: no significance). **(C)** Huh7/MT-Keima cells treated with baicalein, CCCP, and starvation medium as described in (B) were analyzed by confocal microscopy as shown in **(A)** and quantified. The data represent the mean ± SEM (n=10, ***P<0.001, NS: no significance). **(D)** Selected frames obtained by live imaging of 0.5 mM baicalein-treated Huh7/MT-Keima cells are shown. A movie showing the live imaging is shown in Supplemental Video S8. The gradual appearance of the MT-Keima signal after longer wavelength excitation is indicated by white arrowheads.

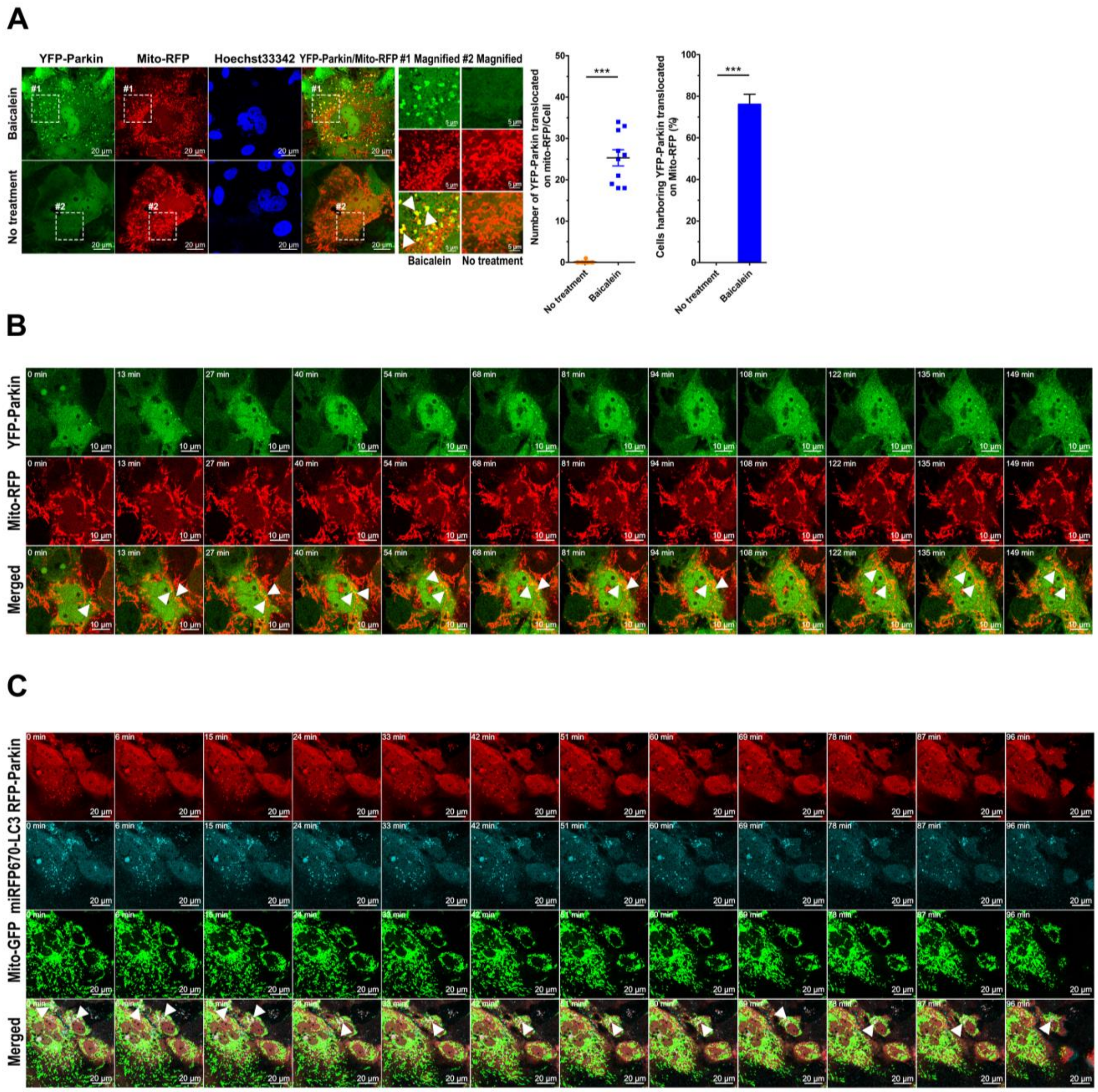

Supplemental Figure S11. Translocation of Parkin to mitochondria in baicalein-treated cells: **(A)** Huh7 cells coexpressing YFP-Parkin and Mito-RFP (Huh7/YFP-Parkin/Mito-RFP) were established by lentiviral gene delivery as described in the "Materials and Methods". The cells were treated with or without 0.5 mM baicalein for four hours, fixed, stained with Hoechst 33342, and analyzed by microscopy. The number of YFP-Parkin translocated into Mito-RFP was quantified. The data represent the means ± SEM (n=10, ***P<0.001). The percentage of cells containing RFP-Parkin translocated into the Mito-GFP was quantified and the data represent the means ± SEM of three independent experiments (***P<0.001). Magnified field-1 and magnified field-2 show enlarged images of the areas in white dashed boxes 1 and 2 in the images of baicalein-treated and untreated cells, respectively. The white arrowheads in magnified field-1 indicate the overlapping signal. **(B)** Selected frames obtained by live imaging of 0.5 mM baicalein-treated Huh7/YFP-Parkin/Mito-RFP cells (Supplemental Video S10) are shown. The translocation of YFP-Parkin to Mito-RFP-labeled areas is indicated by white arrowheads. **(C)** Lentiviruses harboring RFP-Parkin, Mito-GFP, and miRFP670-LC3 were transduced into Huh7 cells, generating Huh7/RFP-Parkin/Mito-GFP/miRFP670-LC3 cells. The baicalein-treated Huh7/RFP-Parkin/Mito-GFP/miRFP670-LC3 cells were analyzed by time-lapse live-cell imaging. Selected frames in Supplemental Video S11 are shown. The white arrowheads indicate the recruitment of miRFP670-LC3 to the RFP-Parkin-translocated Mito-GFP area.

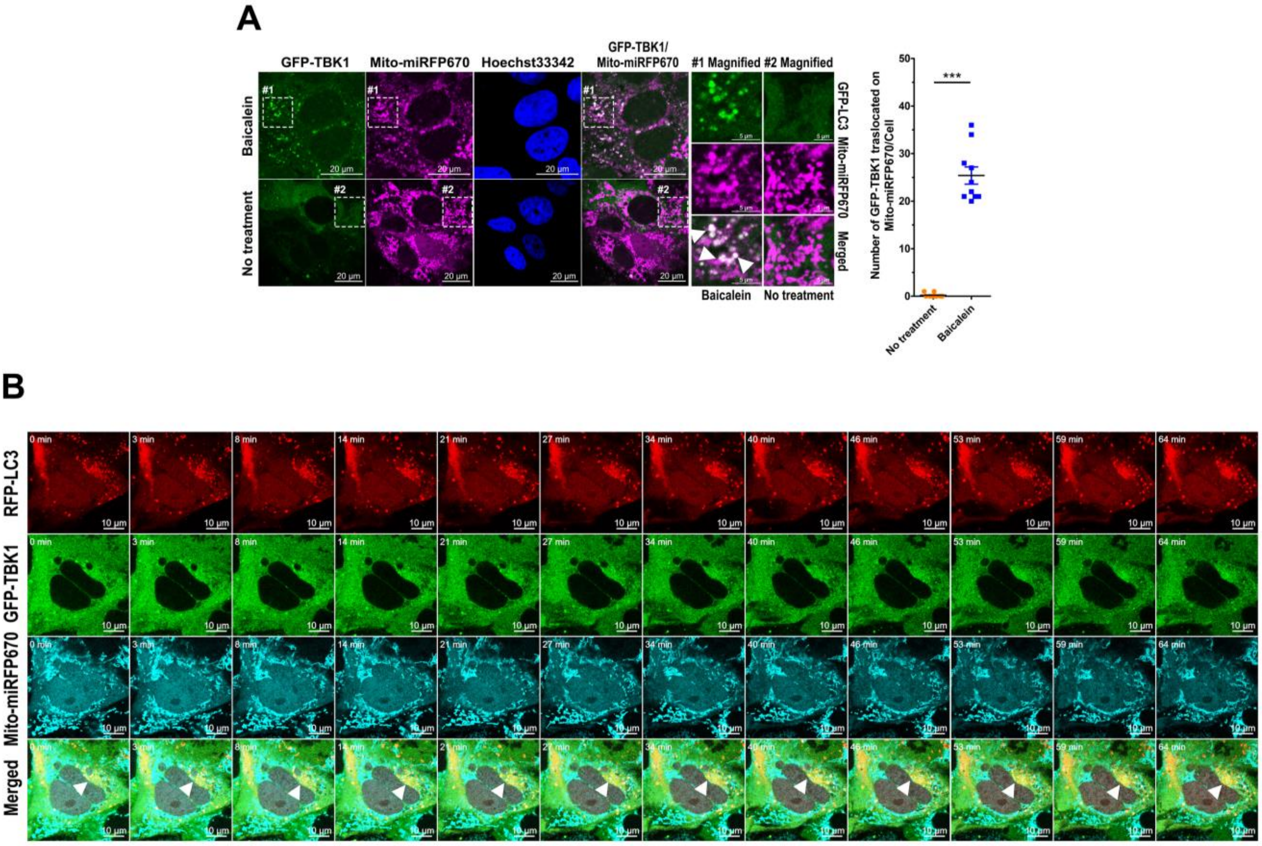

Supplemental Figure S12. Mitochondrial translocation of TBK1 in baicalein-activated mitophagy: **(A)** Huh7/GFP-TBK1/Mito-miRFP670 cells established by lentivirus transduction as described in "Materials and Methods" were treated with or without 0.5 mM baicalein for four hours, fixed and stained with Hoechst 33342, and analyzed by confocal microscopy. The number of GFP-TBK1 translocated on the Mito-miRFP670 shown was quantified. The data represent the means ± SEM (n=10, ***P<0.001). Magnified field-1 and magnified field-2 show the enlarged images of the areas in white dashed boxes 1 and 2 in the images of baicalein-treated and untreated cells, respectively. The white arrowheads in magnified field-1 indicate the overlapping signal. **(B)** Huh7/RFP-LC3/GFP-TBK1/Mito-miRFP670 cells treated with 0.5 mM baicalein were subjected to time-lapse live-cell imaging. The selected frames in Supplemental Video S12 are shown. The representative image with white arrowheads indicates the translocation of GFP-TBK1 during engulfment of Mito-miRFP670 by RFP-LC3 puncta-labeled autophagic vacuoles.

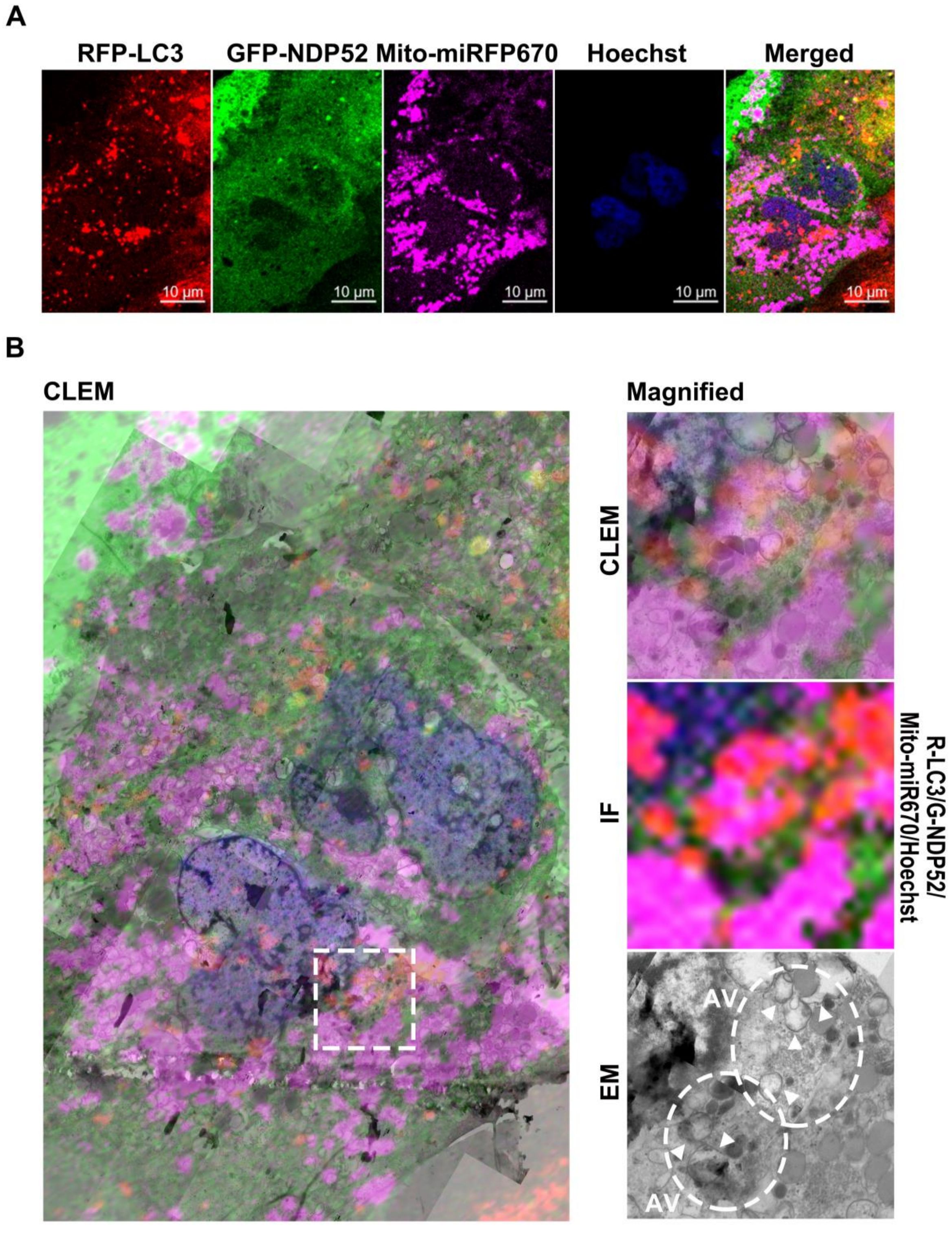

Supplemental Figure S13. CLEM ultrastructural analysis of NDP52-dependent mitophagy in baicalein-treated cells: **(A)** Huh7/RFP-LC3/Mito-miRFP670/GFP-NDP52 cells were treated with 0.5 mM baicalein and analyzed by confocal microscopy. **(B)** The aligned images from IF and EM in CLEM are shown in the left panel. The white dashed box indicates the engulfment of Mito-miRFP670 by RFP-LC3 puncta-labeled autophagic vacuoles associated with GFP-NDP52. The magnified images in the right panel show the enlargement of the area in the white dashed box of the left panel. The white dashed circles and the white arrowheads indicate AV and engulfed mitochondria, respectively.

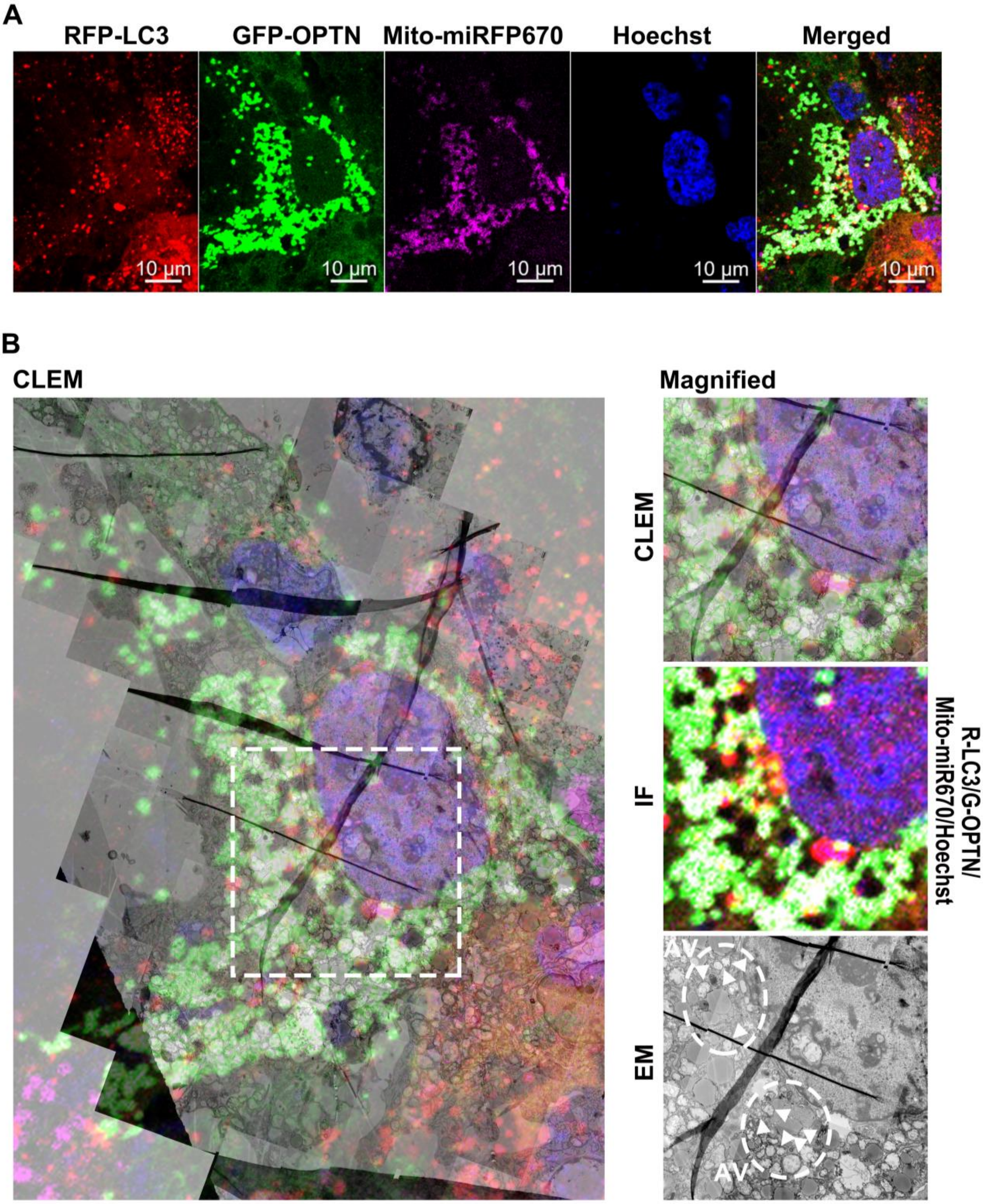

Supplemental Figure S14. CLEM ultrastructural analysis of OPTN-dependent mitophagy in baicalein-treated cells: **(A)** Huh7/RFP-LC3/Mito-miRFP670/GFP-OPTN cells were treated with 0.5 mM baicalein and analyzed by confocal microscopy. **(B)** The alignment of IF and EM images in CLEM is shown in the left panel. The white dashed box shows the sequestration of Mito-miRFP670 within the area in which RFP-LC3 puncta and GFP-OPTN signals merge. The magnified images in the right panel show the enlargement of the area in the white dashed box of the left panel. The white dashed circles and the white arrowheads indicate AV and sequestrated mitochondria, respectively.

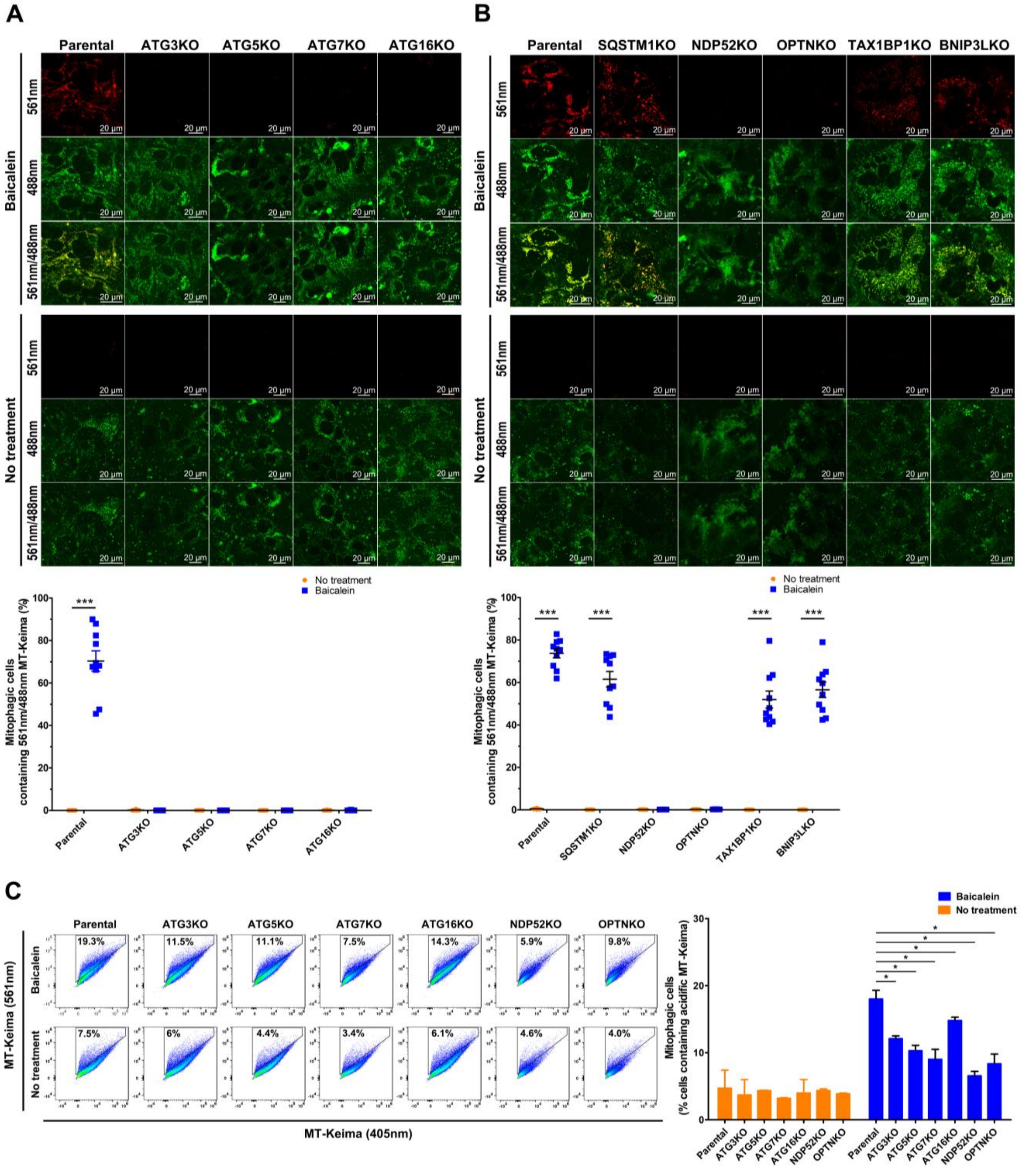

Supplemental Figure S15. Effects of interference with the gene expression of ATGs and cargo receptors on baicalein-activated mitophagy: **(A)** Huh7 parental and individual ATG-KO cells stably expressing MT-Keima established as described in Supplemental Figure 10A were treated with or without 0.5 mM baicalein. Four hours later, the cells were analyzed by confocal microscopy. The number of mitophagic cells containing acidic MT-Keima was quantified as shown in Supplemental Figure 10C. The data represent the mean ± SEM (n=10, ***P<0.001). **(B)** Huh7 parental and individual cargo receptor-KO cells stably expressing MT-Keima were cultured in the presence or absence of 0.5 mM baicalein for four hours. Then, the cells were analyzed by confocal microscopy. The data represent the mean ± SEM (n=10, ***P<0.001). **(C)** Huh7 parental, individual ATG-KO, NDP52KO, and OPTNKO cells were treated with or without 0.5 mM baicalein for four hours and subjected to FACS analysis of MT-Keima expression with excitation wavelengths of 405 nm and 561 nm. The percentage of mitophagic cells in each sample was quantified by FlowJo software and is shown in the right panel. The data represent the mean ± SEM of two independent experiments (*P<0.05).

Supplemental Table 1. sgRNA sequences used for CRISPR/Cas9 gene editing

Gene name	sgRNA sequence (5′ to 3′)
ATG3	TGTTTGCACCGCTTATAGCA
ATG5	AAGAGTAAGTTATTTGACGT
ATG7	GAAGCTGAACGAGTATCGGC
ATG16	TTCCAGGGATGATGACATTG
SQSTM1	AATGGCCATGTCCTACGTGA
NDP52	GATGAGGATGGTGTGGTCCG
OPTN	GCCAGTGGAGACTGTTCTCG
TAX1BP1	CAAGAGGGGACTCTGACAGG
BNIP3L	TCAGGACAGAGTAGTTCCAG
